# A bibliometric analysis of cash holdings literature: current status, development, and agenda for future research

**DOI:** 10.1007/s11301-021-00213-0

**Published:** 2021-03-09

**Authors:** Saleh F. A. Khatib, Dewi Fariha Abdullah, Ernie Hendrawaty, Ahmed A. Elamer

**Affiliations:** 1 Azman Hashim International Business School, Universiti Teknologi Malaysia, 81310 Johor Bahru, Malaysia; 2 Faculty of Economic and Business, Universitas Lampung, Bander Lampung, Lampung, 35141 Indonesia; 3 Brunel Business School, Brunel University London, Kingston Lane, Uxbridge, London, UB8 3PH UK; 4 Department of Accounting, Faculty of Commerce, Mansoura University, Mansoura, Egypt

**Keywords:** Cash holdings, Financial policy, Corporate governance, Payout policy, Corporate social responsibility, Bibliometric analysis

## Abstract

Despite the growing interest in exploring the cash holding aspects among scholars, systematic reviews and comprehensive evaluation in this area has been limited. Also, there is only a fragmented understanding about how the cash holdings concept is formed among researchers and experts. We fill this gap in the literature by identifying and evaluating the research development of cash holdings topic. Using 874 articles from the Scopus database that were published between 1947 and early 2020, bibliometric and content analyses were employed to assess the patterns of global cash holdings research. We find that previous studies have substantially enriched our knowledge of the antecedents and consequences of cash holdings. Yet, there are still several opportunities to make significant contributions in this area. The contribution of this research is to provide a comprehensive evaluation of the development of cash holdings research (using a sizeable archival database). It identifies the current joint development and potential opportunities for future work directions on cash holdings association with payout policy, corporate social responsibility, and corporate governance. Our results are likely to be of interest to academics, practitioners, and educators in related business and finance fields.

## Introduction

Cash holding constitutes a significant aspect at the heart of corporates’ financial policy. Indeed, holding cash is the most popular way for companies to maintain enough liquidity (Almeida et al. 2014). In the past two decades, corporates around the globe have substantially increased their cash holdings as it enables companies to respond to unpredictable cash flow changes, hedge risk, daily financing operations, and financing long-term investments (Opler 1999). Opler (1999) conducted the earliest pioneering empirical study on the causal factors of cash holdings, inspiring the emergence of the scholar interest to this topic. Thus Far, previous studies have substantially enriched our knowledge of the antecedents of cash holdings. However, despite the growing interest in cash holdings topics, there remains a paucity of research on evaluating and describing scientific publications from an international viewpoint (Da Cruz et al. 2019).

The cash management activities of corporations remained an interesting topic among scholars. The cash holding literature has traditionally concentrated on the presence of the target cash holding and its determinants. Companies are more likely to maintain cash for several motives. It allows corporates to avoid losses of underinvestment because of funds scarcity and to diminish transaction costs (Opler 1999). Considering financial instruments, cash holding can be used to finance the firm's operational activities during financial difficulties (Campello et al. 2011). It also helps in reducing the costs of external financing (Almeida et al. 2004), and it serves to pay the obligations of debts during economic distress (Acharya et al. 2007), it also enables corporates to accept profitable investments opportunities (Ferreira and Vilela 2004). Moreover, companies are said to bypass valuable investment opportunities, particularly when facing financing constraints, therefore, cash can be utilized to cover future shortfalls (Bates et al. 2009). These findings were further supported by Almeida et al. (2004), who argue that firms with more financial constraints are more likely to incorporate savings from incremental cash flows to protect their futures. Consequently, hedging for downturns, such firms would keep a substantial amount of cash.

However, holding cash is not costless, and it has been argued that holding a significant amount of cash leads to lower return on investments (Dittmar et al. 2003), because excessive cash is said to be misused by executives (Jensen 1986). Executives might invest in projects with negative net present value due to the conflicts of interest emerge from the ownership separation of corporate. Such investments lead to the agency problem of high cash and reduce the value of their shares (Denis 2001; Jensen 1986). Additionally, holding excessive cash comes with other costs (transaction costs) like flotation fees and taxation affecting the valuation of reserved cash (Faulkender and Wang 2006).

In the cash holdings literature, a stream of researchers focused on the antecedents of cash holding (Akben-Selcuk and Sener 2020; Opler 1999). These research studies further support the individuality of organizational policies and practices of companies' cash holdings that differ across countries because of the differences in the business environments (Tahir and Alifiah 2015). Also, several essential motives were introduced that contribute to our understanding of the antecedents and outcomes of cash holding such as agency motives, transaction motive, and precautionary motive.

The agency motives for holding cash posit that due to the differences of interest between owners and executives, when executives might not act in the best interests of stockholders, they may use the company's excess cash for their wealth maximization instead of serving the owner (Jensen 1986). Hence, entrenched executives are more likely to hold more cash to maximize their personal benefits. Lee and Lee (2009) provide empirical evidence on the association between managerial entrenchment and cash holdings to be positively significant.

Transaction motive indicates that a company firm needs liquid resources to finance its daily operation and in case of scarcity of cash at a time when needed, a company might have to liquidate assets to meet its obligation, sometimes it will have to pay transaction costs. These costs can be avoided by keeping more liquid assets (Drobetz and Grüninger 2007; O’ Brien and Folta 2009). The precautionary motive emerges from the asymmetric information impact on external funds raising. In the future of a firm, there are unexpected additional expenses like price fluctuations, rising costs, and availability of raw materials, or any other unpredicted situations. In such scenarios, cash holding helps to cover the companies to meet these needs (Kawase et al. 2015; Ozkan and Ozkan 2004; Xu et al. 2019). However, it should be noted that previous studies have substantially enriched our knowledge about cash holdings. Yet, a little is known about the outcomes of cash holding (Jebran et al. 2019). Also, a limited number of researches have focused on evaluating and describing scientific publications from an international viewpoint (Da Cruz et al. 2019), Pointing to a need for comprehensive research to evaluate and review cash holdings topics from a broad sample of studies.

The limited number of researches have focused on evaluating and describing scientific publications from an international viewpoint (Aliyev et al. 2019; Block et al. 2020; Block and Fisch 2020; Da Cruz et al. 2019), while other review studies have devoted a significant focus on the determinants of cash holdings (He 2018; Tahir and Alifiah 2015; Weidemann 2018). Similarly, Amess et al. (2015) concentrate on the determinant of cash holding and its association with corporate governance. Also, Akhtar et al. (2010) have limited their review work to the association between corporate governance and cash holding. Furthermore, a systematic analysis study conducted by Da Cruz et al. (2019) to explore the development of cash holding studies by using several databases for data mining. However, their study was limited to articles published in the journal with impact factor one or above excluding a large number of articles from the analysis (Table 1 summarizes the key differences between these review studies and the current study). Although there is extensive work on cash holdings explorings the association with other aspects of corporations, core debates on cash holdings remain to be addressed (Da Cruz et al. 2019). We believe that this field has reached the level of maturity which necessitates an in-depth and comprehensive evaluation of the cash holding researches. We also argue that previous review studies are better to be integrated with research that includes a comprehensive overview to map the existing knowledge on cash holding topic (Castriotta et al. 2019). Such work helps to consolidate the achievements of the field and craft a research agenda for years to come.

**Table 1:** A comparison of our study and similar research on cash holdings

Author	The focus of the study	Sample	Methodology
Weidemann (2018)	Determinants of cash holding, and theories used in the literature	Not specify	Content analysis
Amess et al. (2015)	Corporate governance, cash holding, and determinants of cash holding	Not specify	Content analysis
Da Cruz et al. (2019)	Determinants cash holdings, the value of cash holdings, precautionary, transaction cost, the sensitivity of cash to cash flow and or to investment, motives of excess cash, and agency conflicts	A string of keywords 144 articles 1997–2017	Systematic review of journals with an impact factor of 1 and above
Akhtar et al. (2010)	Review of the association between cash holding and corporate governance	Not specify	Critical review
Our research	Cash holding (all aspects)	A string of keywords 874 articles 1947–2020	Content, structured review, and bibliometric analysis

By using a large number of articles, this research addresses several questions related to cash holdings: (i) What is the current publication trend of cash holdings research? (ii) What is the leading articles, countries, authors, and journals in term of the publication or 'citations' number? (iii) Which topics involving cash holding are the most recent or common among scholars? (iv) what is the intellectual development of the field? and (v) What themes involving cash holding needs more attention from researchers?

This study has used the Scopus database for data mining. Many similar studies in various fields including management have been conducted using this database only (Drago and Aliberti 2019; Md Khudzari et al. 2018; Moreira et al. 2019; Yahaya et al. 2020; Zheng and Kouwenberg 2019). It should be noted that Scopus data covers a wide range of subjects, and it is the most significant citation/abstract database (Md Khudzari et al. 2018). Following Da Cruz et al. (2019) the central theme in this research was all journal papers that cover the following terms in the title and abstract “Cash*Holding*”. Data collection was conducted in May 2020 and the query search string has resulted in 874 documents published from 1947 to 2020. This large number of sample literature enables us to map the development and contribution of the cash holdings studies and identifying challenges and avenues for future studies on this topic.

This paper contributes to the existing literature about cash holdings by presenting a comprehensive review of the existing studies. This study provides a review of the research landscape in the area of cash holdings and presents interesting insights and directions for future research. This paper also contributes to the theoretical development of cash holdings research because it helps researchers discover possible opportunities and determine the key research themes in cash holdings literature (Shi and Li 2019). Our study departs from previous reviews studies by being the first to provide a combination of systematic literature review and bibliometric analysis on cash holdings, offering a complementary approach to the more traditional literature review. Such criteria are based on a previous systematic literature review (Kumar and Ranjani 2019; Moreira et al. 2019; Baker et al. 2020), and it has been proven to be useful to academicians in identifying the current research structure of the subject and will inform them about the evolution of the various themes in this area (Kumar et al. 2020). This paper contributes to existing debates about determinants of cash holdings and suggest that country-level aspects exert a significant role determining the level of cash holdings. Also, it has been found highlighting that agency, trade-off, and theories are the dominating theoretical aspect in this area, yet, the interplay of cash holding theories is not well understood (Weidemann 2018).

The remainder of the paper is structured as follows. Section 2 explains the methodology employed in this research including the analysis methods and the searching strategy. Section 4 discusses the findings of the study. Section 5 summarizes several topics related to cash holdings that have been attracting researchers’ interests. Section 6 Conclusion of the research.

## Methodology

Bibliometric analysis can be defined as the structured process of describing all documents that have been published in a specific field of science in terms of the number, connection, productivity, quality, citations, and evaluating the intellectual development of the scientific field. Ronda-Pupo (2017) suggested that the research activities of a scientific field can be an excellent tool to understand its structure. However, Block and Fisch (2020) suggested that providing only a list of references (leading research, authors, institutions, etc.) followed by a brief description does not qualify the research to be a bibliographic study, a bibliographic study should also focus on evaluating the structure of a particular research field (Block and Fisch 2020). Therefore, we followed the instructions provided by the novel work of Block and Fisch (2020) to conduct an impactful bibliometric study that evaluates the development of research on cash holdings. This process would help us understand the development of a scientific field.

Unlike review papers that mainly focus on discussing the latest progress, future directions, and challenges of a specific topic, the focus of this study is two folds. First, this study employed a bibliometric type of analysis to evaluate the research development on cash holding as it is an effective method to address the research trends on a particular topic by exploring existing documents (Md Khudzari et al. 2018; Shi and Li 2019). Second, following the systematic review method of Moreira et al. (2019) to conduct a rigorous bibliometric and content analysis of several themes that emerge from keywords and citations clusters. To identify the intellectual structure of the research on board diversity, citation and co-citation analysis were performed using VoSviewer. The co-citation network was formed using VosViewer to present the thematic flow of knowledge and the formation of clusters. Lead papers from the clusters were identified using weighted citation measure and then were used to perform cluster analysis. These methods enable us to provide a comprehensive evaluation of the development of cash holding research from an international perspective.

**Fig. 1:**
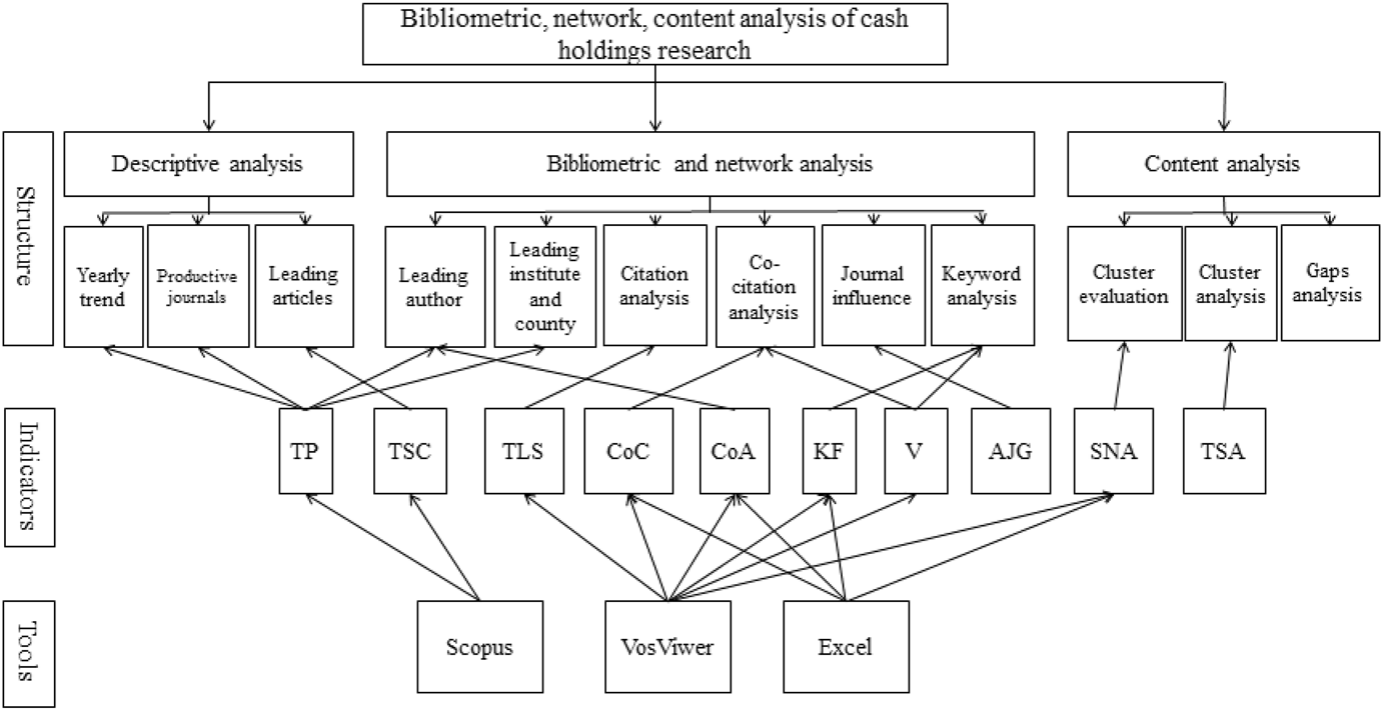
Analytic structure of this paper. Note; TSA thematic structure analysis, SNA social network analysis, AJG academic journal guide, V vissualization view, KF keyword frequency, CoA co-authorship, CoC co-citation count, TLC total local citation, TSC total Scopus citation, TP total publications

To map the literature, we employed VOSviewer as it is a powerful software tool to construct a visualized map based on the link to the objects of interest. This software has been widely used in bibliometric research investigation (Behrend and Eulerich 2019 [auditing]; Castriotta et al. 2019 [emerging organization structure]; Zheng and Kouwenberg 2019 [corporate governance]; Yahaya et al. 2020 [Innovation]). VOSviewer maps were created in this study for keywords of authors, document citations, and authors' network as they are the key concern of this study. The methods used in the analytical structure of this study are presented in Fig. 1. It also shows the organization of this study, calculation, indicator, and the tool used.

### Defining searching terms

Block and Fisch (2020) suggested that it is important for bibliometric studies to have a clear, transparent, reproducible searching process, this section is, therefore, presenting the process of data mining in a clear manner. Data collection was conducted in May 2020 from the Scopus database. Many similar studies in various fields including management have been conducted using Scopus database only (see, Md Khudzari et al. 2018; Drago and Aliberti 2019; Zheng and Kouwenberg 2019; Yahaya et al. 2020). It should be noted that Scopus data covers a wide range of subjects, and it is the most significant citation and abstract database and it is the most commonly used search databases (Amrutha and Geetha 2020; Md Khudzari et al. 2018). The advantage of this database is that it allows researcher to import a bibliography database for all the final results including citation matrix, publisher, affiliation, references, etc. in a single excel (.CSV) file. After reviewing the similar publication, definitions, and categories of cash holding, we developed a central theme search string. Given that cash holding is a broad topic, unlike Da Cruz et al. (2019), we limited our search by using the primary theme keyword. The central theme in this research was all journal papers that include 'Cash* Holding*' terms in the titles, keywords, and abstracts.

### Search delimiting criteria

**Fig. 2:**
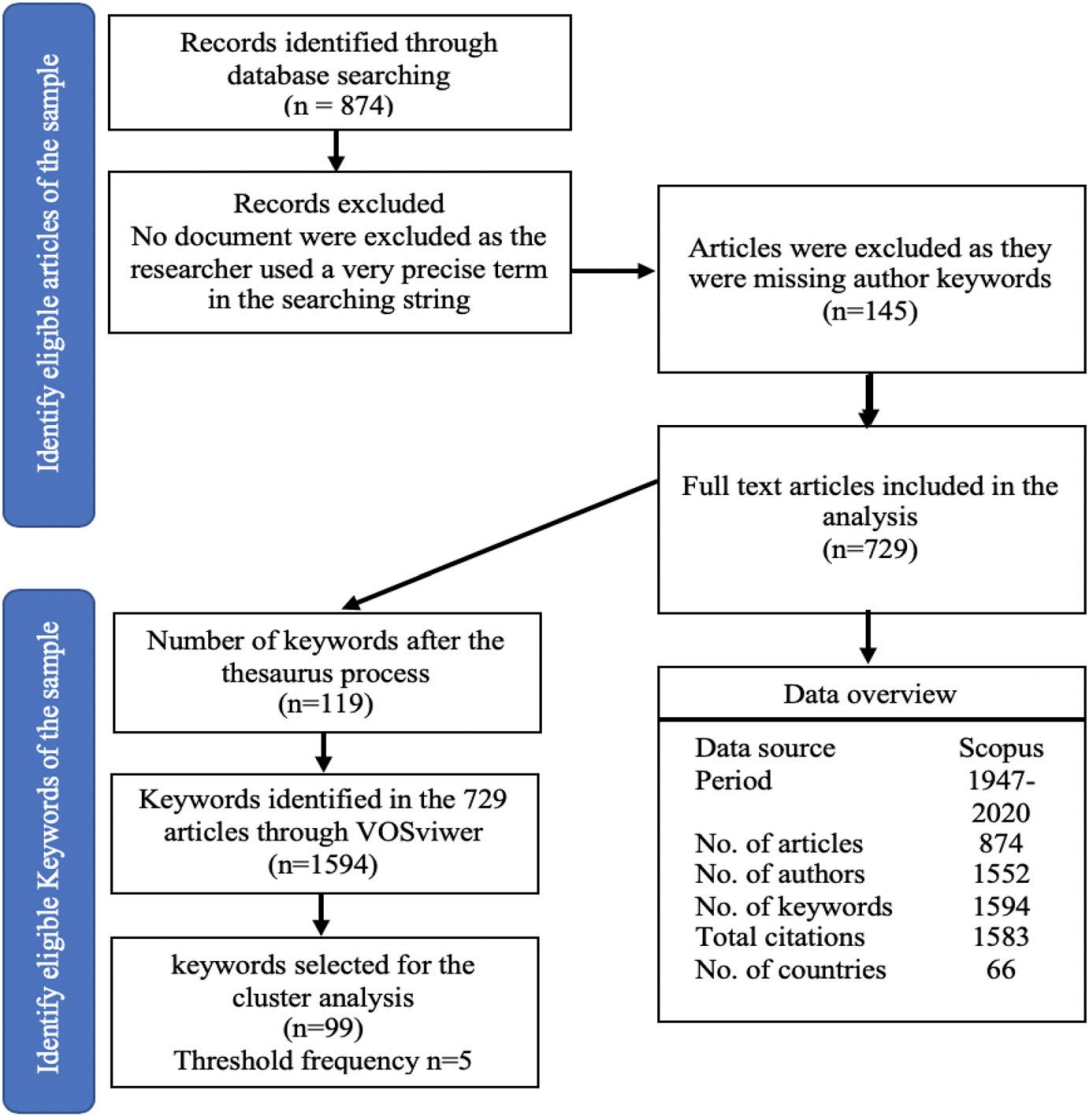
Workflow diagram for conducting bibliometric research

The query string has resulted in 874 documents published from 1947 to 2020 in the Scopus database after limiting the search for a journal article that is published in the English language. In the descriptive analysis (country/territory, affiliations, authors, sources, and years), all articles were utilized. For the keywords analysis, following the method of Md Khudzari et al. (2018) and Yahaya et al. (2020), 145 articles were excluded from the keyword analysis due to the lack of the keywords. This approach leaves us with 729 articles (1594 keywords) to map the development of the cash holdings studies and identifying challenges and avenues for future studies on this topic (see, Fig. 2). For the content analysis, this study follows the method of Moreira et al. (2019) to identify the themes of content analysis. Four themes were subject to the content evaluation in cash holding primary output. Two of them emerged from the cluster analysis (corporate governance and determinants of cash holdings) and the other two emerged from the keyword analysis, namely payout policy and corporate social responsibility. This is depicted in Table 5 later.

## Descriptive analysis

A descriptive analysis of 874 articles was carried out to know the basic ongoing trend of publication on this topic. To achieve the first research question, we have first analyzed the publication trend, which is seen in terms of total publication by year, country, region, journal, and institution.

### Growth of the publication

**Fig. 3:**
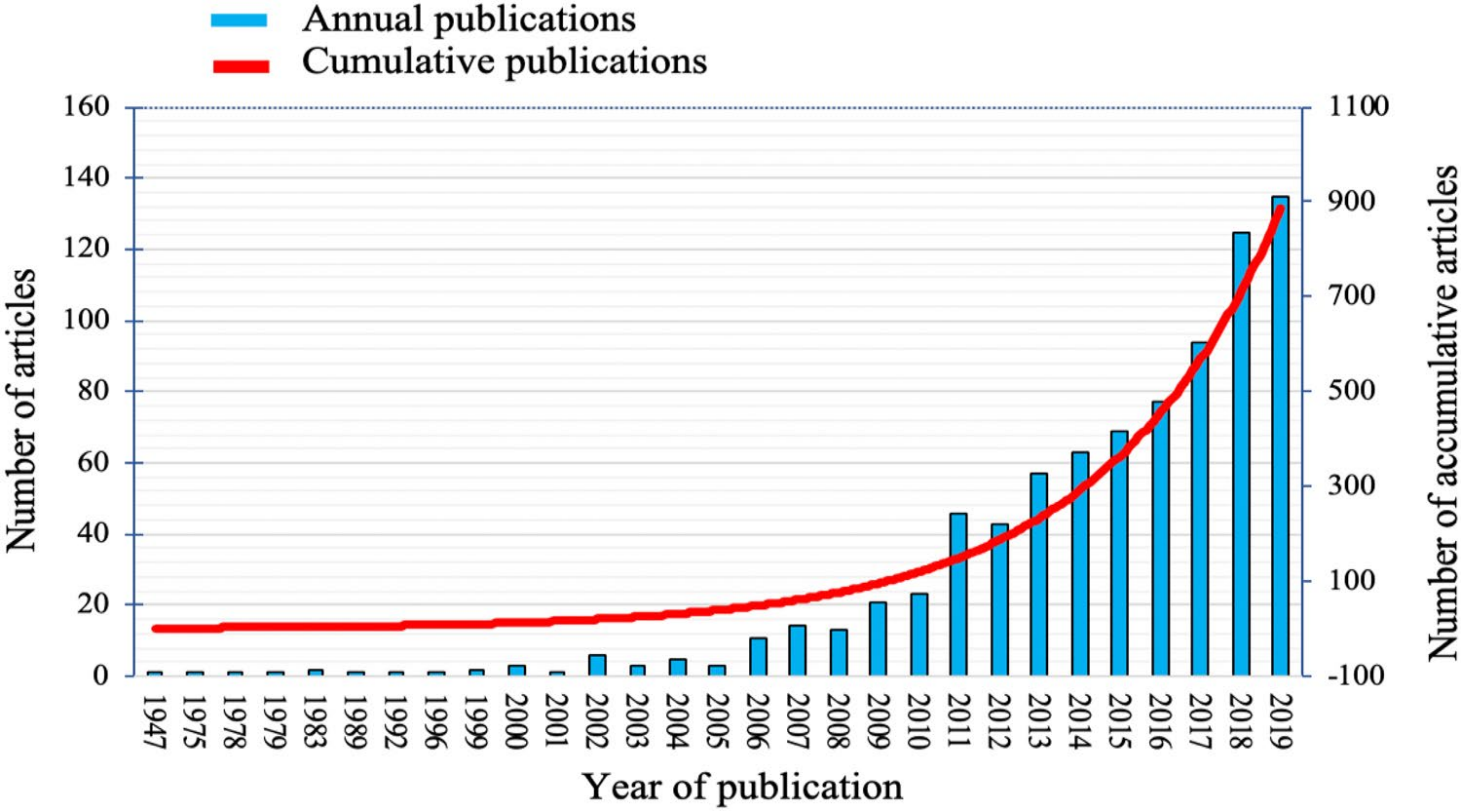
The cumulative and annual numbers of publications on cash holding

874 documents had been published in Scopus indexing journals related to cash holdings. The oldest document dates to 1947, and there were no publications recorded until 1975. Eleven articles have been published before 2000. The number of annual publications remained below 100 articles in total until 2010. At first glance, it is clear that a keen interest in the cash holdings topic started in 2010–2011 (see, Fig. 3). One possible explanation is that both developed and emerging economies have significantly affected by the international economic crisis in 2007. This downturn has triggered scholars to explore more the financial behaviour of corporations around the globe, including cash holding. Indeed, literature has stressed that the economic downturn has affected the cash holding practices of corporations (Jebran et al. 2019). Also, Tran (2020) found evidence that during the economic downturn time, executives were more likely to expropriate stockholders through the firm's liquidity policy. Hence, it is expected to see more work on cash holdings in the recent future evaluating the Covid-19 impact on the different aspects of cash holding. In 2010, there was a sharp increase in publications and it was almost doubled in 2011. Later, the accumulative number of documents has rapidly increased as a result of the steady growth in the number of annual articles. However, it should be noted that the majority of these publications are not free and readers have to pay for access. Out of 874, only 85 documents were open-access publication. It has been suggested that an open-access research paper more likely receives more citations than other journal articles.

### Journals outlets

The findings indicate that only four publisher agencies owned the top 10 most productive journals (Table 2). Five of them were from Elsevier, including the four most productive journals. Wiley-Blackwell published two journals. The rest were published by Springer Nature, Cambridge University Press, and Oxford University Press. Furthermore, the most productive one was the Journal of Corporate Finance with 58 articles covering 6.6% of the total publications, followed by the Journal of Banking and Finance (32, 3.7%). Journal of Financial Economics (31, 3.5%) published the most cited paper among the top ten with 982 citations by Opler (1999). The rest of the journals were counted for less than 2.5% of total publications each. Moreover, based on the CiteScore in 2019, the number of journals with CiteScore 5 and above are three. Interestingly, although the rank of Journal of Finance is the 9th with 15 documents, the CiteScore and total citation were the highest in the list (8.30). The Journal of Financial Economics is the second-highest CiteScore (7.67). Indeed, the Journal of Financial Economics publishes four of the most cited articles.

**Table 2:** The most productive journals on cash holdings publication and the most cited documents

Journal name	T.P	T.C 2019	CiteScore 2019	The most cited document	Time cited	Publisher
Journal of corporate finance	58	1018	4.11	Han and Qiu (2007)	174	Elsevier
Journal of banking and finance	32	801	3.22	Ozkan and Ozkan (2004)	271	Elsevier
Journal of financial economics	31	3965	7.67	Opler (1999)	982	Elsevier
International review of economics and finance	20	73	2.48	Kuan, Li and Liu (2012)	33	Elsevier
Accounting and finance	19	161	2.66	García-Teruel et al. (2009)	29	Wiley-Blackwell
Review of financial studies	18	987	6.42	Denis and Sibilkov (2010)	280	Oxford University Press
Review of Quantitative Finance and Accounting	16	93	1.95	Hill et al. (2014)	19	Springer Nature
Journal of Financial and Quantitative Analysis	16	823	4.43	Dittmar et al. (2003)	406	Cambridge University Press
Journal of Finance	15	2520	8.30	Bates et al. (2009)	652	Wiley-Blackwell
International Review of Financial Analysis	13	69	3.05	Amess et al. (2015)	18	Elsevier

^*^T.C. total citations, *T.P.* total publications

## Bibliometric analysis

### Leading countries and institutions

This study shows that more than 50% of the total publications on the cash holdings topic were contributed by the USA and China which indicates that these countries are key players in the development of this topic (Table 3). The USA led the list with 329 publications, covering 38% of the total global publications. National Bureau of Economic Research is the most productive institution in the USA with total publications of 14 documents. It should be noted that it is also the most productive institution on cash holdings research. Among the 14 countries, two countries only seemed to have more than two-thirds of a single-country publication, namely Japan (81%), and Malaysia (85%). This indicates a strong collaboration between countries. The country with the least SCP was Hong Kong (16%); out of 24, about 20 documents were affiliated to different countries. Furthermore, the investigation revealed that few studies taking place in the African region, Latin America, and the Middle East countries while the main body of literature mostly and repeatedly collected data from developed countries.

**Table 3:** The top 15 prolific academic institutions and countries in cash holdings topic

Country	Total publication	Single country publication	The most prolific institution	Total publication by the institution
USA	329	204 (62%)	National Bureau of Economic Research	14
China	129	76 (59%)	Peking University	9
UK	92	45 (49%)	Centre for Economic Policy Research, London	10
South Korea	48	31 (65%)	Korea University	8
Taiwan	47	30 (64%)	National Chung Cheng University	7
Australia	46	19 (41%)	Monash University	7
Canada	45	15 (33%)	University of Toronto	9
France	33	12 (36%)	Université Paris-Est	7
Spain	29	19 (65%)	Universidad de Salamanca	8
Germany	28	13 (46%)	Universität Hamburg	7
Hong Kong	24	4 (16%)	City University of Hong Kong	10
India	23	15 (65%)	Monash University	3
Japan	22	18 (81%)	Osaka University	4
Malaysia	21	18 (85%)	Universiti Utara Malaysia	7

### Leading authors

We find that the ten most productive authors in cash holdings are affiliated with seven countries (Table 4). The total number of documents of these authors were counted for 48 documents. It indicates a high collaboration between them. Additionally, Chen, N. from Taiwan, is the most productive author with a total of 7 documents since 2007, 28 citations, and four h-index. Similarly, Lozano, M.B. from Spain got the same number of publications (7) and only nine citations by the end of 2019. The 3rd and 4th top authors are Drobetz W. from Germany and Ozkan N. from the U.K. with six articles each. Interestingly, Pinkowitz, L. is the least productive author within the top ten. Yet, he has the highest citation record.

**Table 4:** List of the ten most productive authors in cash holdings research topic

Author's name	Year of first publication	TP	H-index	TC 2019	Affiliation	Country
Chen, N	2007	7	4	28	National Chung Cheng University	Taiwan
Lozano, M. B	2012	7	2	9	Universidad de Salamanca	Spain
Drobetz, W	2007	6	4	128	Universität Hamburg	Germany
Ozkan, N	2004	6	5	372	School of Economics, Finance, and Management	The U.K
Boubaker, S	2014	5	3	52	Université Paris-Est	France
Chan, K.C	2012	5	2	52	Western Kentucky University	The USA
Derouiche, I	2014	5	3	52	University of Luxembourg	Luxembourg
Harford, J	2008	5	5	771	Foster School of Business	The USA
Maxwell, W.F	2007	5	5	783	SMU Cox School of Business	The USA
Pinkowitz, L	1999	5	5	1493	McDonough School of Business	The USA

^*^TC total citations. *TP* total publications

### Journal influence and quality analysis

To evaluate the journal influence in cash holdings research, we applied two methods. We assess leading journals in the area of cash holdings using analyzed the average citation per article (ACA) as an indicator. Then, we evaluate the Academic Journal Guide (AJG) rating of the journals. This analysis would help to differentiate between the quality and productivity of journal publications. As the number of citations is an indicator of the journal influence while the number of publications is an indicator of journal productivity. Following Kumar and Ranjani (2019), we calculated the ACA of the leading journals based on the total citations from Scopus database. As it is shown in Table 5, although the Journal of Finance has 15 publish documents on cash holdings, it has the highest ACA within the list, followed by the Journal of Financial Economics. This interesting finding indicates that a large number of publications do not always guarantee more citations as the case with the Journal of Finance.

**Table 5:** Average citation per article of top journals

Journal name	Total publications	Total citations 2019	ACA
Journal of corporate finance	58	1018	17.6
Journal of banking and finance	32	801	25.0
Journal of financial economics	31	3965	127.9
International review of economics and finance	20	73	3.6
Accounting and finance	19	161	8.5
Review of financial studies	18	987	54.8
Review of quantitative Finance and Accounting	16	93	5.8
Journal of financial and quantitative analysis	16	823	51.4
Journal of finance	15	2520	168
International review of financial analysis	13	69	5.3

**Fig. 4:**
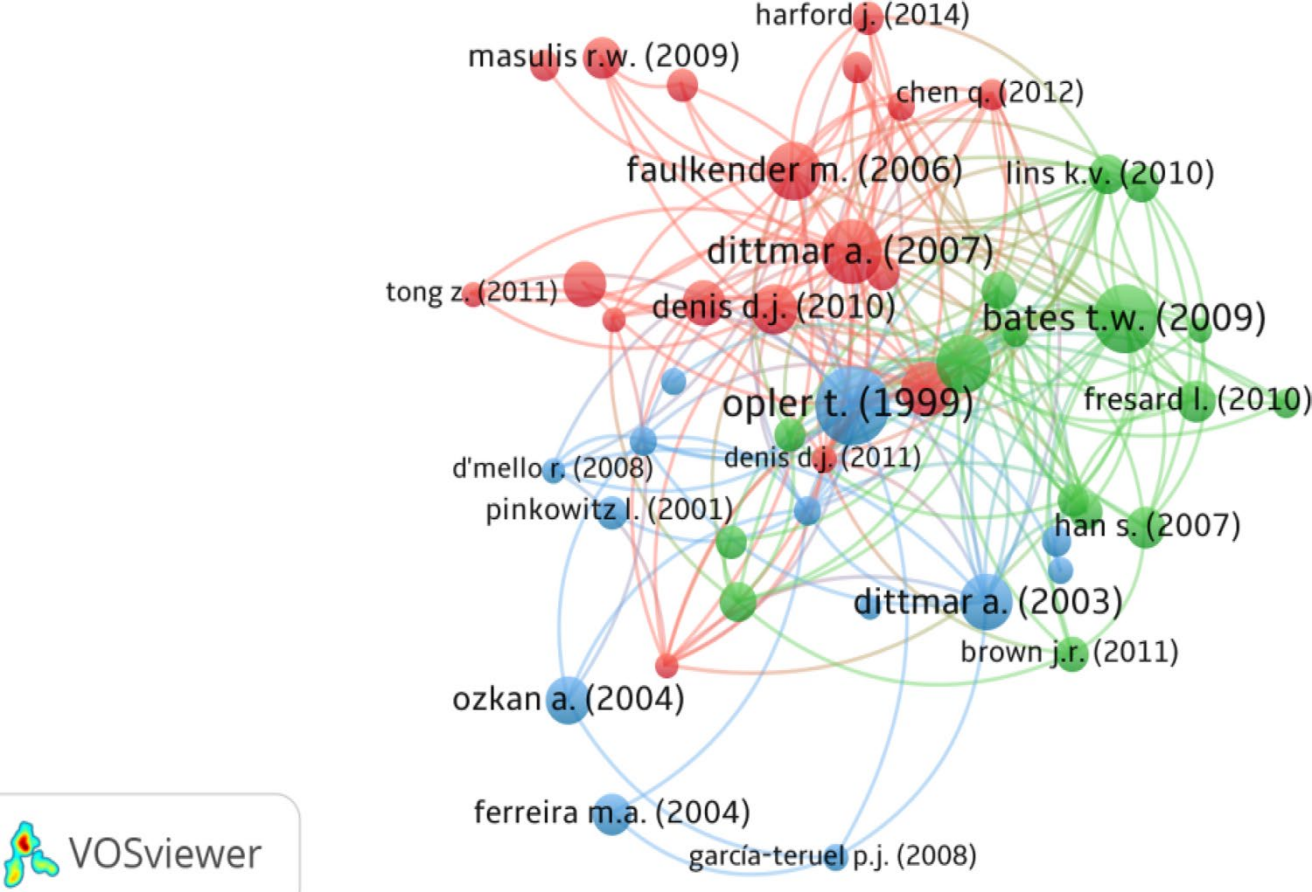
Map of documents citations 50 articles (minimum of 60 citations)

For journal quality analysis, the Academic Journal Guide 2018 was also utilized to evaluate the quality of the studies. It provides a quality ranks of the journals in management and business and categorizes them into the given groups 4*, 4, 3, 2, and 1. One is the lowest quality while 4* is the highest. AJG rates are an important tool for researchers' promotions in business schools and commonly utilized by scholars (Kumar and Ranjani 2019). The results indicate that a vast majority of cash holdings research has been published in none ranked journal (212 articles). Interestingly, out of 874 documents, only 73 (4.2%) articles have been published in Grade 4* journals. As it is shown in Fig. 4, researchers were interested in Grade 2 journals, followed by Grade 3 and 4 with 219, 193, 96 articles respectively.

### Citation analysis

We used citation analysis to identify the most popular articles within the cash holding community. It has been suggested that citation analysis counts the number of times other articles cite a particular article to identify the popularity and impact of the article in the scientific field (Kumar et al. 2020). Based on the based on ‘total times cited count’ provided by the Scopus database, we have analyzed the citation of 874 studies. The findings of the documents’ citations suggest the most cited articles were published in five different journals (Table 6). The first one was conducted by Opler (1999) with 982 citations as it is considered to be among the first studies to examine the determinants of cash holdings, followed by Bates et al. (2009) with 652 citations. However, among the most cited papers on cash holding topic, four articles were published by the Journal of Financial Economics and three published by the Journal of Finance. Interestingly, the AJG assessment shows that eight of the top ten leading studies are from the 4% of cash holding documents that are published in Grade 4* journals discussed in the previous section.

**Table 6:** The most cited articles on cash holdings

Rank	Authors	Citations 2019	Journal	AJG
1	Opler (1999)	982	Journal of Financial Economics	4*
2	Bates et al. (2009)	652	Journal of Finance	4*
3	Dittmar et al. (2007)	592	Journal of Financial Economics	4*
4	Harford et al. (2008)	465	Journal of Financial Economics	4*
5	Barclay and Holderness (1989)	464	Journal of Financial Economics	4*
6	Faulkender et al. (2006)	436	Journal of Finance	4*
7	Dittmar et al. (2003)	407	Journal of Financial and Quantitative Analysis	4
8	Pinkowitz et al. (2006)	367	Journal of Finance	4*
9	Denis and Sibilkov (2010)	280	Review of Financial Studies	4*
10	Ozkan and Ozkan (2004)	272	Journal of Banking and Finance	3

### Citation trend

We analyze the references of our sample literature (874 documents) and this analysis results in 31,018 unique references that were cited by the sample literature. Out of 31,018, 3225 articles were locally cited two times or more. The locally cited document refers to the number of citations for an article within our sample of literature. Following Kumar and Ranjani (2019), we used this analysis as another means of evaluating the most influential studies within the cash holdings community. As shown in Table 7, we found that there are eight research articles with more than 50 local citations. Opler (1999) is the highest in the list with 163 local citations and 48 links indicating that this study has been mentioned 163 times in the reference lists of our sample literature. Dittmar and Mahrt-Smith (2007) who studied the association between cash holdings and governance structure, were cited by about 15% of the cash holdings literature. Additionally, Dittmar et al. (2003) conducted a study investigating the correlation between investor protection and cash holding in 45 countries which was slightly less cited in the sample literature (89 times). The results indicated that about five percent of the cash holding studies have cited Faulkender and Wang (2006), Harford (1999), Han and Qiu (2007), Myers and Majluf (1984), and Almeida et al. (2004). However, this investigation provides use with the first glance on the thematic trend of cash holdings research. After reviewing these studies, we conclude that the thematic series following in cash holdings research is not wide and concentrated on corporate governance, financial policy, and the determinants of cash holdings.

**Table 7:** Documents with over 100 local citations

Article	The focus of the study	Total links	Local citations
Opler (1999)	Factors that determine the level of cash holdings in companies	48	163
Dittmar and Mahrt-Smith (2007)	The association between cash holdings and governance structure	47	138
Dittmar et al. (2003)	Shareholder protection and cash holdings	54	89
Faulkender and Wang (2006)	The corporate cash holdings, marginal value, and financial policy	38	78
Harford (1999)	Corporate acquisitions and cash holdings	45	72
Han and Qiu (2007)	The precautionary motive for a firm's cash holdings	55	63
Myers and Majluf (1984)	Corporate financing behavior and pecking order theory	31	62
Almeida et al. (2004)	The financial constraints and firms' cash flow	36	57

### Co-citation analysis

Co-citation also helps to structure literature and the thematic clusters and gaps in the scientific area (Block and Fisch 2020). It refers to the occurrence of two references in the reference list of a single document. Co-citation analysis helps identify the content and subject area by evaluating the more frequently cited reference together. The occurrence of two publications more than one time on the reference list of an article can be an indicator of the similarity in empirical discipline, methodology, theory, and topic. We used the link strength between two documents provided by VoSviewer to measure the connection between pair references. As suggested by Van Eck and Waltman (2013) this measure for each pair of linked items and indicates the strength of their connection. The co-citation investigation found that there exist 201 pairs of documents that are co-cited with each other at least 10 times. Among these 201 connections, the strongest co-citation connection exists between Dittmar and Mahrt-Smith (2007) and Opler (1999); the link strength between these articles is 78. Table 8 presents the pairs of authors with the highest number of link strength. As indicated in the table, the second strongest co-citation connection exists between Dittmar and Mahrt-Smith (2007) and Dittmar and Mahrt-Smith (2007), followed by Faulkender and Wang (2006) and Dittmar and Mahrt-Smith (2007). It should be noted that the highest connection list is concentrated among Opler (1999), Dittmar and Mahrt-Smith (2007), Dittmar et al. (2003), Faulkender and Wang (2006), Harford (1999), Han and Qiu (2007), Myers and Majluf (1984), and Ozkan et al. (2004). This finding confirms our discussion in the earlier section where the body literature of cash holdings is focusing on the determinant of cash holdings, corporate governance, and financial policy of corporations.

**Table 8:** Document pairs with more than 30 link strength

No	Author 1	Author 2	Total link strength
1	Opler (1999)	Dittmar and Mahrt-Smith (2007)	78
2	Dittmar and Mahrt-Smith (2007)	Dittmar et al. (2003)	61
3	Faulkender and Wang (2006)	Dittmar and Mahrt-Smith (2007)	59
4	Dittmar et al. (2003)	Opler (1999)	56
5	Harford (1999)	Dittmar and Mahrt-Smith (2007)	43
6	Faulkender and Wang (2006)	Opler (1999)	41
7	Harford (1999)	Opler (1999)	36
8	Opler (1999)	Han and Qiu (2007)	35
9	Opler (1999)	Myers and Majluf (1984)	33
10	Opler (1999)	Ozkan et al. (2004)	31
11	Harford (1999)	Dittmar et al. (2003)	30

#### Co-citation network and data clustering

To address the intellectual development of the field, the co-citation network analysis was applied. From the co-citation network, several clusters were identified to conduct the content analysis. To study the intellectual structure of the topic ‘cash holdings’, we start by using VosViewer for the co-citation network analysis. Co-citation analysis in VosViewer gave us a.TEXT file, which we used in Excel to read the co-citation network. The initial findings result in 3225 references that are at least twice co-cited with one another. Among them, 65 articles occurred together more than 20 times. To visualize the co-citation map, VosViewer formed a random cluster map that was too complex to understand. Therefore, we follow Kumar and Ranjani (2019), who identified the leading 10 papers from each cluster. Similarly, we used the weighted citation count provided by VosViewer to ensure high-quality papers in cluster analysis.

**Fig. 5:**
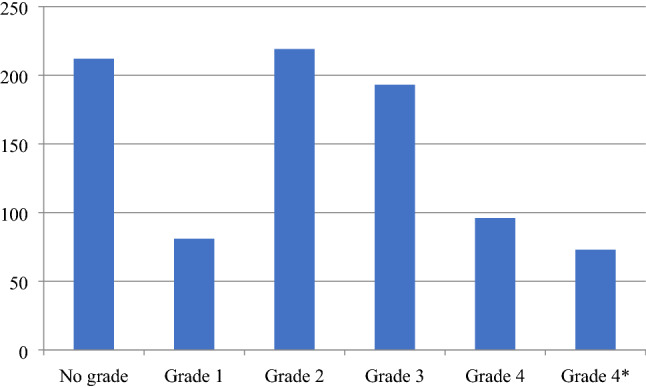
Academic Journal Guide (AJG) 2018 rating of 874 articles

As shown in Fig. 5, the analysis results in three clusters with a high correlation between them. Among the three clusters, the red group is the largest that is led by Dittmar and Mahrt-Smith (2007). Followed by the green cluster with Bates et al. (2009) as the most dominant study. Finally, the blue cluster is dominated by Opler (1999), the most cited author in cash holdings literature. It should be noted that despite that the fact that these clusters address different aspects of cash holdings, they are highly interrelated and complement each other. Furthermore, following Moreira et al. (2019), we pooled these clusters based on the topic covered by each study with reference to the cited articles over 3 per cluster. As a result, two main themes related to cash holdings emerged: corporate governance, the determinant of cash holdings. These themes were then grouped and analyzed in terms of the association with cash holdings, followed by a summary of each article. The literature about cash holdings is still unclear about its determinants and the impact of corporate governance. This is depicted in Table 7 and 8 later.

##### Group 1: corporate governance and cash holdings

The corporate governance mechanisms quality is the central influencing factor of corporate financial policies, including cash holding (Abdelfattah et al. 2020; Abdou et al. 2020; AlHares et al. [Bibr CR1002][Bibr CR60]; Alnabsha et al. 2018; Alshbili et al. 2019; Alshbili and Elamer 2020; Hazaea, Zhu, et al. , b; Khatib et al. 2020). Corporate governance practices are expected to ensure that executives act in the best interest of stockholders (Asante-Darko et al. 2018; Bufarwa et al. 2020; El-Dyasty and Elamer 2020; Elamer et al. 2018, 2020, 2019; Elamer and Benyazid 2018; Elmagrhi et al. 2018; Hazaea et al. 2020a). Jensen (1986) stressed that executives are reluctant to disburse the extra cash among stockholders to secure their benefits by investing it in unprofitable projects, which might destroy corporates' valuation. As a result of the ownership separation, the self-interested executive more likely to exploit the firm assets to maximize their personal wealth at the expense of stockholders' interest (Jensen and Meckling 1976). This detrimental effect of extensive cash saving on corporate performance is canceled in well-governed companies (Dittmar and Mahrt-Smith 2007; Luo and Hachiya 2005). Table 9 provides a summary of all studies in cluster one.

**Table 9:** Cash holdings and corporate governance

Author	Focus	Method	Findings summary
Dittmar and Mahrt-Smith (2007)	Corporate governance and the value and the use of cash holdings	1,952 firms 1990 to 2003 The USA OLS regression	The cash is dissipated quickly in poor governance companies which leads to poor performance
Harford et al. (2008)	Corporate governance and the management of cash holdings	1,872 firms Cross-country 1993–2004 Multivariate Analysis	The cash reserves are smaller in weaker governance firms. Also, they spend the cash primarily on acquisitions rather than investing internally
Chen et al. (2015)	Managerial expropriation	1,340 firms Cross-country 1999 to 2011 OLS	CEOs receive a high excess compensation in firms experience an exogenous decrease in analyst coverage, and management is more likely to make value-destroying. Also, shareholders value internal cash holdings less
Chen et al. (2012)	Cash holdings and corporate governance in the context of the split share structure reform	1,293 firms China 2000–2008 Difference-in-differences method	The firms' cash management policies are significantly affected by the governance reform including capital structure, dividends payout, and investment decision. This impact is different in state-owned and private companies
Liu and Mauer (2011)	Cash holdings and CEO compensation incentives	20,439 firm-years The USA 1992–2006 2SLS Probit	There is a negative association between CEO risk-taking and the value of cash to shareholders. While there is a positive relationship between cash holdings and CEO risk-taking incentives
Pinkowitz et al. (2006)	Investor protection	12,339 firms cross-country 1983–1998 Fama–Macbeth OLS	The relationship between firm value and the dividend is weak in strong investor protection countries In a market with poor investor protection, the association between firm performance a cash holding is weak
Masulis and Mobbs (2011)	Inside directorship	2,137 firms Cross-country 1997–2006 Difference-in-difference, 2SLS, and OLS	Companies make better acquisition decisions, have greater cash-holdings and overstate earnings less often if inside directors holding outside directorship
Custódio and Metzger (2014)	CEOs experience	4,277 CEOs Cross-country 1993–2007 OLS	CEOs with financial experience manage financial policies more actively, less likely to use one companywide discount rate instead of a project-specific one, and they are better to obtain external funds
Dittmar et al. (2003)	Shareholder protection and cash holdings	11,000 firms Cross-country 1998 OLS regression	In markets with poor investor protection, firms hold cash twice as much as firms in markets where the shareholders' rights are well-protected. Also, investment opportunities and asymmetric information are less important in poor shareholder protection countries

Corporate governance appears to be the most explored topic in relation to cash holding as evident by the number of keyword occurrences (96 times). Studies on this association can be categorized into several groups: first, studies that concentrated on board of director characteristics (Asante-Darko et al. 2018; Atif et al. 2019; Boubaker et al. 2015; Roy 2018; Thanatawee 2019). The overall evidence of the cash holding pattern of corporate governance negative (Harford et al. 2008; Roy 2018). The agency literature suggests that management can be prevented from holding excessive cash by the high quality of governance mechanisms like better law enforcement and higher investor protection (Da Cruz et al. 2019). Similarly, Dittmar and Mahrt-Smith (2007) reported that poor-governed companies dissipate cash quickly and in ways that significantly reduce operating performance. However, the findings of prior studies are still inconclusive. For instance, Dogru and Sirakay-Turk (2018) contend that cash holding is more considerable in well-governed corporates than in poorly governed ones. While Akben-Selcuk et al*.* (2020) argue that the structure of boardroom does not exert a significant influence on the level of cash holdings. Apart from the mixed findings, some mechanisms have been overlooked in the literature such as board diversity (Khatib et al. 2021). The existing research on board diversity and cash holding has concentrated on gender diversity as an indicator of board diversity, neglecting other indicators like ethnic, educational, experience, age, tenure, and others (see, Atif et al. 2019; Cambrea et al. 2019). Atif et al. (2019) reported a significant negative association between more gender diverse boardroom and cash holdings. Whereas, more diversity in terms of educated tend to keep extra cash (Wang et al. 2017).

The second school concentrated on the association between ownership structure and cash holdings. Amess et al. (2015) suggest that any governance mechanisms that able to mitigate the agency conflicts, including ownership structure, results in reducing the cash holding in firms. It is well documented in the literature that the ownership structure has a significant impact on cash holding. Among different ownership structures, managerial ownership has received large attention among cash holding scholars (Chen and Chuang 2009; Drobetz and Grüninger 2007; Lee and Lee 2009; Ozkan and Ozkan 2004; Thanatawee 2019; Yu et al. 2015). Supporting the incentive-alignment hypothesis, Thanatawee (2019) found that the level of cash holding is lower as the managerial ownership increase indicating that executives do not keep extra cash for personal benefits. While Yu et al. (2015) suggest that a higher level of firm cash holding is associated with a higher percentage of managerial ownership supporting the argument that board monitoring and managerial incentives are substitutes for each other. Others reported a non-linear association between cash holding and managerial ownership, indicating an incentive alignment effect and an opposing effect related to increased risk aversion (Drobetz and Grüninger 2007; Ozkan and Ozkan 2004). Additionally, using institutional ownership as the primary governance indicator, Nguyen and Rahman (2020) reported a negative relationship between cash holdings and corporate governance. A similar finding was documented by Loncan (2018) after focusing on foreign institutional ownership, suggesting that this effect is potentially transmitted to cash by reducing the agency problems and alleviate the financial constraint. Moreover, scant attention has been given to other ownership attributes like ownership concentration and directors ownership (see, Ferreira and Vilela 2004; Taufil Mohd et al. 2015).

Third, we found that there is a limited but recent interest among scholars in evaluating the impact of chief executive officer (CEO) characteristics on cash holding (average publication per year 2017.42). Chief Executive Officer (CEO) characteristics are known to affect corporate financial policies (Intintoli and Kahle 2016). Orens and Reheul (2013) argue that it is essential for shareholders to account for the demographics of current or future CEOs and to know their associated preferences concerning cash policy. However, they limited their study to the age and experience of the CEO and found a significant influence on the cash holding of small-medium enterprises. Furthermore, Liu et al. (2014) suggested a positive relation between CEOs deferred compensation (in debt) and firm cash holdings. Zeng and Wang (2015) examine the CEO’s gender effect on cash holding and found that the female CEOs are related to a higher level of corporates cash saving. While Yung et al. (2015) suggested that CEOs overconfidence do not have a significant impact on cash holdings. However, despite the growing number of studies on the impact of corporate governance, ownership structure, and CEO characteristics on cash holdings, the findings of prior research result in mixed conclusion and some attributes have received a little attention from research such as ownership concentration, board diversity, and CEO demographic characteristics (Feng et al. 2020; Hassan et al. 2020; Khatib et al. 2020; Malagila et al. 2020; Roberts et al. 2020). Thus, we believe that research on this area remains a fertile ground for future research.

##### Group 2: The determinants of cash holdings

The finance literature has paid more attention to the determinants of firm cash holding (Jebran et al. 2019). Understanding the factors behind holding cash in corporations, offers a critical insight into the complexity of cash management and finance decisions (Tahir and Alifiah 2015). After the pioneering empirical study of Opler (1999) who conducted the earliest research on the determinants of cash holdings inspiring the emergence of the scholar interest in this topic, the determinants of cash holdings witnessed a gradual increase in the number of studies. Table 10 provides a summary of all studies on the determinants of cash holdings resulting from the cluster analysis (cluster 2). The literature has suggested that public firms have high level of cash holding than private one due to the agency costs and conflict (Gao et al. 2013). This is in line with Ferreira and Vilela (2004) who found that concentrated ownership and investor protection are important factors affecting the level of cash in corporations. Also, several additional factors have been proven in the literature to be significant in determining the level of cash holdings, such as credit risk, which has been found to exert a positive impact on cash level (Acharya et al. 2012; Opler 1999). Similar relation was reported with dividend payments, cash conversion cycles, investment opportunity, and firm size (Bigelli and Sánchez-Vidal 2012; Ferreira and Vilela 2004; García-Teruel and Martínez-Solano 2008; Opler 1999). In contrast, there is an adverse relationship between bank debt, leverage, asset’s liquidity, the interest rates in the economy, concentrated ownership, investor protection, capital markets development, and cash holdings (Ferreira and Vilela 2004; García-Teruel and Martínez-Solano 2008). It should be noted that these studies have mainly focused on the firm-level determinants, while country-level aspects have been less investigated.

**Table 10:** The determinants of cash holdings

Author	Focus	Method	Findings summary
Gao et al. (2013)	Cash holdings in private and public firms	7,879 firms The USA 1995–2011 OLS regression	The cash holdings of public firms are twice as much as private firms because of the higher agency costs in public firms. Agency conflict is a significant driver of the target level of cash
Opler (1999)	Determinants of cash holdings	87,117 firm-year observations The USA 1971–1994 OLS	The cash level is higher in companies with riskier cash flows and strong growth opportunities. While this level is lower if companies with high credit ratings and large corporates because these firms have better access to the capital markets. The occurrence of operating losses is the underlying factor for the significant changes in the cash level. Well-performed firms tend to hold cash more than the predicted level
García-Teruel and Martínez-Solano (2008)	Determinants of cash holdings in of SMEs	860 firms Spain 1996–2001 OLS, and GMM	Companies with more bank debt and when the interest rates in the economy increase, firms tend to hold have a low target level of cash holdings. While this level increase in companies with larger cash flows and more growth opportunities
Lins et al. (2010)	The driver of firms’ liquidity	204 CFOs Cross-country Logit	Credit and non-operational (excess) liquidity are used to hedge against different risks. In good times, credit lines give firms the option to exploit future business opportunities, while non-operational cash guards against future cash flow shock in bad times
Pinkowitz and Williamson (2001)	The effect of bank power on cash holdings	1971–1994 Cross-country OLS regression	The monopoly power of banks exerts a significant impact on Japanese cash balances. The cash levels in Japanese firms are similar to U.S. firms if the bank’s power is weakened
Bates et al. (2009)	The motives behind cash holdings	13,599 firms The USA 1980–2006 OLS	Firms in great idiosyncratic volatility sectors, more recent IPO listing cohorts, and do not distribute dividends experience an increase in the cash ratio. The reasons for this growth is R&D expenditures and cash flow risks have increased, capital expenditures and inventories have fallen
Ozkan and Ozkan (2004)	Determinants of cash holdings	1,029 firms The UK 1984–1999 GMM estimator	Cash holdings are significantly influenced by the managerial ownership. This influence is different based on the control concentration. The key determinants of cash holdings are bank debt, leverage, liquid assets, cash flows, and growth options of firms
Ferreira and Vilela (2004)	Determinants of cash holdings	6,387 firm-year Cross-country 1987–2000 OLS	The cash flows and the investment opportunity exert a positive impact on cash holdings. In contrast, there is an adverse relationship between bank debt, leverage, asset’s liquidity, concentrated ownership, investor protection, capital markets development, and cash holdings
Bigelli and Sánchez-Vidal (2012)	Determinants of cash holdings	17,165 firms Italy 1996–2005 GMM	Networking capital and bank debt represent good cash-substitutes. Dividend payments are associated with more cash holdings. Lower effective tax rates, higher risk, and small size exert a significant impact on cash holdings. The level of cash is higher in companies with lower financing deficits and longer cash conversion cycles
Brown and Petersen (2011)	Cash holdings and R&D smoothing	57,845 The USA 1970–2006 GMM	Firms use cash holdings extensively to smooth R&D if they face financing frictions
Acharya et al. (2012)	Cash holdings and credit risk	24,496 The USA 1996–2003 OLS and logit	The precautionary savings are central to understanding the effects of cash on credit risk. There is a positive association between the optimal cash level and credit risk

There is a growing body of literature highlighting the firm-level aspects as important cash holding determinants such as risk (Acharya et al. 2012; Bates et al. 2009; Bigelli and Sánchez-Vidal 2012; Opler 1999), growth opportunity (García-Teruel and Martínez-Solano 2008; Opler 1999), bank debt (García-Teruel and Martínez-Solano 2008; Ozkan and Ozkan 2004), R&D expenditures (Bates et al. 2009; Brown and Petersen 2011; Dittmar et al. 2003), and size (Bigelli and Sánchez-Vidal 2012; Ozkan and Ozkan 2004). Furthermore, studies have examined the determinants of cash holdings in different levels of investor protection markets (Ferreira and Vilela 2004), and industries (Bates et al. 2009). However, it should be noted that the literature about the determinants of cash holdings still unclear in some contexts. For example, Opler (1999) suggested that there is an inverse relationship between firm size and the level of cash holdings in the USA, while Ozkan and Ozkan (2004) used a large sample from the UK and found this association to be insignificant.

## Keywords analysis

The keywords co-occurrence is an insightful technique to investigate scientific constructs according to the presumption that keywords provide a coherent explanation to the content of the documents (Comerio and Strozzi 2019). The connection between two keywords presented by a numerical value which shows the relationship between both of them, and the higher this value, the stronger the link (link strength). The link strength between two keywords represents the number of times where these keywords occurred appeared in the same article. The total number of these links refers to the aggregate number these two keywords occur together. In VOSviewer, five were set as the minimum occurrences of a keyword to be presented, which means that keywords will appear on the bibliometric map once two keywords occur together in a document more than five times.

The keywords co-occurrence analysis conducted in this study involved 1594 keywords from 729 articles. Because of a lack of keywords in 78 journals, the other 145 documents were omitted from the analysis of the keywords. Also, synonymic keywords were analyzed before inserting the data into VOSviewer. For instance, “the number of cash holdings,” “cash holding (ch)”, “cash holding balances”, “cash holdings”, and “cash-holding levels” were re-labelled as “cash holdings” and counted as one keyword. The same re-labelling process was conducted for all other keywords.

However, a large number of these keywords have been used once. About 1248 (78.3%) were used one time, 176 keywords (11%) were used two times, and 51 (3.2%) was used three times. The number of total keywords decreased to 1199 by re-labelling them. The keywords were inserted in VOSviewer to map the literature with a minimum of 5 occurrences, and only 99 keywords met the thesaurus.

**Fig. 6:**
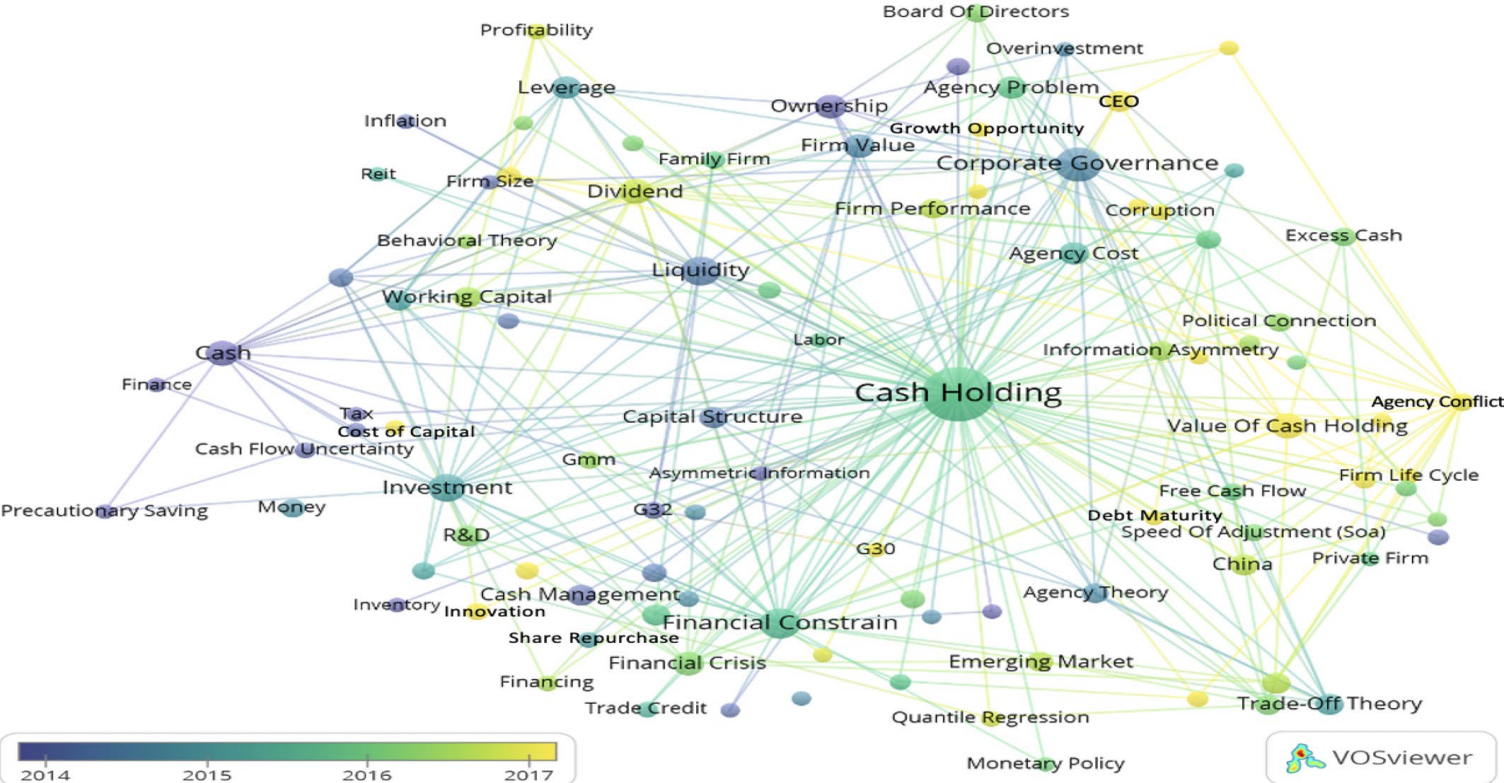
The bibliometric map of author keywords co-occurrence. To open this map in VOSviewer, use the following URL: https://bit.ly/2XLu2eD

It has been suggested that the keywords co-occurrence analyses are representative enough to make general claims about the articles' content (Comerio and Strozzi 2019). Scholars usually employ co-occurrence analyses as it is an effective method to address the research trends on a particular topic by exploring existing documents (Md Khudzari et al. 2018; Shi and Li 2019), including the field of economics and finance (Castriotta et al. 2019). Following Baker et al. (2020) and Md Khudzari et al. (2018), we conduct keyword co-occurrence to evaluate the prevalent themes within cash holding. The result from the keywords co-occurrence map (Fig. 6) shows that cash holding research is mainly focused on corporate governance (96 occurrences, 66 links strength). It indicates that most of the researches on cash holdings concentrated on examining the association between corporate governance mechanisms and cash holding. One possible explanation for this is that the financial policy of corporates is significantly affected by the corporate governance quality and the agency conflicts among agents and principles (Lee and Lee 2009).

Additionally, given that cash holding is considered as one approaches a firm would follow to structure its capital, it was expected to have a strong association with other financial aspects such as “financial constrain” which is second most topics examined concerning cash holding (67 occurrences, 43 link strength). Furthermore, liquidity (55 occurrences) was noted to be also highly related to cash holding (30 link strength). We also encountered general terms such as ‘investment’ (46 occurrences, 47 links), 'cash" (36 occurrences, 28 links). Table 11 provides a summary of the most frequent keywords.

**Table 11:** Top keywords by the frequency of their occurrence

Keyword	Frequency	Keyword	Frequency
Cash Holding	443	Cash flow sensitivity	19
Corporate Governance	96	CEO	19
Financial constrain	67	Working capital	19
Liquidity	55	China	18
Investment	46	Precautionary motive	18
Cash	36	Capital structure	17
Dividend	33	R&D	17
Value of cash Holding	31	Cash flow	16
Financial crisis	27	Emerging market	16
Firm value	26	Firm performance	16
Ownership	26	Pecking order theory	16
Agency cost	24	Agency theory	15
Agency problem	21	information Asymmetry	14
Leverage	21	Acquisitions and mergers	13
Cash management	20		
Trade-off theory	20		

The colour in Fig. 6 shows the average year of publications in which the keyword has occurred, overlay visualization mode with five as minimum occurrences. We also illustrate the most recent and oldest extremes of keywords in cash holding in Table 12 with three minimum keyword occurrences. It is noteworthy that, in our unit analysis, taxation, risk management, and initial public offer are the oldest topics to be investigated in relation to cash holding. The interest in the taxation theme has been renewed in recent years but tax avoidance aspects. Additionally, it is worth noting that advanced methodological techniques were introduced in 2015, with terms such as quantile regression and generalized method of moments (GMM). Hence, it is expected in the future to see more work with these methods and others, such as structural equation modelling and difference-in-differences techniques that have been overlooked in the literature.

**Table 12:** The overlay visualization terms composition in terms of the average year of publication

Average year of publication	Themes
2010–2013	Tax (n = 6), Risk management (n = 3), and Initial Public offer (n = 3)
2014	Corporate governance (n = 96), Ownership (n = 26), Firm value (n = 26), Cash management (n = 20), Capital structure (n = 17), Transaction cost (n = 7), Firm size (n = 6), Inflation (n = 6), and Share repurchase (n = 6)
2015	Financial constrain (n = 27), Agency cost (n = 24), Leverage (n = 21), Acquisitions and mergers (n = 13), Cash flow sensitivity (n = 19), Family firm (n = 10), Free cash flow (n = 10), SMEs (n = 9), and Underinvestment (n = 4)
2016	Dividend (n = 33), Precautionary motive (n = 18), R&D (n = 17), Firm performance (n = 16), Political connection (n = 11), Board of directors (n = 12), Speed of adjustment (n = 10), GMM (n = 9), Quantile regression (n = 7), Risk-taking (n = 4), and Risk aversion (n = 4), Audit (n = 3)
2017	Value of cash holding (n = 31), CEO (n = 19), Capital expenditure (n = 10), Innovation (n = 9), Business group (n = 8), Corruption (n = 7), Corruption (n = 7), Firm life cycle (n = 6), Debt maturity (n = 5), Managerial ownership (n = 5), Cost of capital (n = 5), Growth opportunity (n = 5), and Accounting conservatism (n = 3)
2018-present	Economic policy uncertainty (n = 10), Corporate social responsibility (n = 9), Gender (n = 5), Tax avoidance (n = 4), CFO (n = 4), Country governance (n = 3), and Board independence (n = 3)

Furthermore, corporate governance is the most frequent key-term, and scholars are moving toward more specific governance mechanisms such as board independence (3 occurrences; 2018), country governance (3 occurrences; 2018), and board of directors (12 occurrences; 2016). Finally, corporate social responsibility has drawn researchers' attention recently, and it can be a potential hot topic for future studies due to the limited work on this association. From the keywords’ frequency, two topics of interest were selected for the content analysis including payout policy and corporate social responsibility, while corporate governance as the most examined area in relation to cash holdings is discussed in the cluster analysis later. To evaluate these topics, we searched within our literature sample for specific terms that are related to each output separately.

### Topics of interest and thematic evolution

#### Cash holding and payout policy

Payout policies (dividends payment and share repurchase) are common ways for corporations to re-balance their capital structure by either reducing or increasing the cash holdings. The association between cash holding and payout police is well documented in the literature, as evidenced by the number of keyword occurrences. However, there are very few studies on share repurchase and cash holdings (Almeida et al. 2016; Haw et al. 2011; Moon et al. 2015). The majority of researches has focused on dividend as the mean of cash distribution (Jia and McMahon 2019; Kumar and Ranjani 2019; Yang et al. 2020). Yet, the finding of prior studies is still inconclusive. The association between cash holding and dividend payment is suggested to be positive (Jia and McMahon 2019; Kuldeep and Misra 2019). On the other hand, Ahmad and Adaoglu (2019) focus on the determinant of cash holding and find the association between dividend and cash reserving to be negative and these findings were further supported by Lee and Lee (2019). However, Palazzo (2012) stressed that companies tradeoff between the dividends distribution and cash reserve, reaching the optimal cash holding level, which helps firms to lower the costs of external financing.

Scholars start to examine the interaction between different factors in an attempt to solve the puzzle of cash holding. Yang et al. (2020) examine the innovation and payout policy relationship and find that R&D company with higher internal financing deficit and lower cash holding have pay-for-finance incentives where it pays dividends to facilitate the access external financing activities (Yang et al. 2020). Doan and Iskandar-Datta (2020) focus on the gender diversity of top management and its impact on cash holding. They find that corporates with surplus cash and female CFOs distribute more dividends among shareholders. Furthermore, Lee and Lee (2019) examine the interrelationship between research and development intensity, dividend, cash holding, and company value in biotech corporates. They suggest reserve extra cash can be achieved by lowering dividends level, and this cash hoarding is positively linked with the firm valuation in the long-term. At the same time, Jia and McMahon(2019) suggested dividend payments decline in companies where the increase of cash holding is a result of a permanent-growth of in corporate profitability. However, although it is well documented in the literature that the relationship between payout policies and cash holding is significant, the sign of their effects is unclear.

#### Cash holding and corporate social responsibility

The primary concern of corporate social responsibility (CSR) is to achieve the ultimate satisfaction of various stakeholders of corporations. Stakeholders are defined as a different group of individuals engaged in the activities of a business, including competitors, employees, customers, suppliers, creditors, shareholders and society. However, despite the importance of this aspect in today’s business environment, only a handful of research has been carried out to examine the relationship between corporate social responsibility and cash holdings. Yang et al. (2019) investigate the effect of CSR on the cash holding valuation and find that in the capital market, CSR does enhance the cash holdings’ value, and this positive effect increases with the firm's market value. This result supports the conclusion of Arouri and Pijourlet (2017) firms with a high corporate social responsibility rating associated with higher value to cash held from investors. Another study by Lu et al. (2017) dedicated to examining the CSR report relationship with cash holding, suggested that information in CSR reports can facilitate monitoring and thus induce more efficient use of cash holdings. Hence, the issuance of a standalone CSR report increases the marginal value of cash holdings. Lastly, one study identified to examine the association between cash holdings and CSR by Cheung and Wai (2016) suggesting a positive relationship between cash holdings and CSR. To our knowledge and based on the study results, very few studies have been conducted to examine the relationship between corporate social responsibility and cash holdings and as such, this warrants future investigation.

## Conclusion, limitations and further research

Cash holding is considered as a significant aspect at the heart of corporates' financial policy. In fact, cash holding is the most popular mean of maintaining enough liquidity within corporations. In the past two decades, corporates around the globe have substantially increased their cash holding. Consequently, a growing interest of researchers has been devoted to cash holding topics, especially after the global financial crisis of 2007 and it is expected to see more work on cash holdings in the recent future evaluating the Covid-19 pandemic impact on the different aspects of cash holding (Khatib and Nour 2021). Despite the growing number of empirical studies on cash holdings, there is a lack of review work that analyzing and evaluating the cash holding publications from an international perspective and different aspects of this topic.

We have used a bibliometric and content analysis to assess the development of global cash holding research from several aspects. The data used in this study were retrieved from Scopus (874 journal articles in total). 145 articles were excluded from the keyword analysis due to the lack of the keywords. Furthermore, the content analysis was limited to four themes that emerged from the keyword and cluster analysis, namely, corporate governance, determinants of cash holdings, payout policy, and corporate social responsibility.

Overall, the results suggest that the number of articles has been rapidly increasing since the financial crisis. Interestingly, about 50% of the total global publications were contributed by developed countries (the USA, the U.K., and China), while that few studies taking place in the African region, Latin America, and the Middle East countries. We, therefore, encourage scholars to explore more the cash holding topic from emerging countries especially a single-industry research as it has been suggested that industries might differ in terms of liquidity needs and other factors influencing the cash holdings level. Similarly, there is a lack of focus on small-medium firms. Surprisingly, SMEs occurred nine times only, and researchers tend to concentrate on significant industries and firms due to data availability. Therefore, we highly encourage scholars to explore cash holdings topics in different industries and SMEs.

Regarding payout policy, there is a lack of work on share repurchase and cash holding relationship and more research on cash holding and payout policy (dividend and share repurchase) is highly recommended. ﻿We do not recommend merely replicating previous research. Future studies may take advantage of including more socio-economic factors and cultural aspects to extend the understanding of cash holding. Moreover, corporate governance is the most examined topic in relation to cash holdings. Yet, the findings of prior studies are mixed, especially when it comes to different governance attributes. Besides, some mechanisms have been overlooked by scholars such as CEO characteristics, ownership attributes and board diversity in terms of ethnicity, education, tenure, experience, national diversity. Pointing to a need for further work to consider the impact of governance attributes that are less addressed in the literature and the interaction between them. Finally, in the last five years, only a handful of researches have been conducted to explore the association between cash holdings and corporate social responsibility. We, therefore, encourage studies to explore further the influence of cash holdings on CSR, sustainability, and environmental performance/disclosure.

This article contributes to this topic for being the first of its kind to provide a comprehensive evaluation of the development of cash holding research form from the international perspective. By using a large number of articles, this study presents an overview of the research landscape in the area of cash holdings that provides interesting insights and directions for future research offering a complementary approach to the more traditional literature review. Our study underlines the dominating topic related to cash holding (corporate governance) and the recent research trends which would be useful for both academics and professionals. From an application viewpoint, this study will serve as basic groundwork for understanding research into the cash holdings, its current stage and the direction in which the research is growing. Also, it highlights the gap in the current body of knowledge and proposes several actionable avenues for future research in the subject. Indeed, within the concept of cash holdings itself lies the implication that it may affect several organizational aspects, as far as cash holding involves all the companies’ management bodies with decision-making powers.

Like other researches, our literature review has some limitations. First, the search technique used in this study was restricted to cash* holding* within the titles, abstracts, and keywords. However, some research might not refer to the cash holding within the searching scope. Also, we limited our search to the search scope to the Scopus database as it is considered as the most extensive citation and abstract database of peer-reviewed articles. Hence, the finding of the search string used in this study may not cover all publications on cash holding. Further research could make a comparison of the outputs from multiple databases.
